# The Efficacy and Prognostic Factors of the Combination of TACE and Apatinib for the Treatment of BCLC Stage C Hepatocellular Carcinoma

**DOI:** 10.3389/fmed.2021.774345

**Published:** 2021-12-03

**Authors:** Shun Liu, Kai-Cai Liu, Wei-Fu Lv, Dong Lu, Xian-Hai Zhu, Bo Jiang, Yu-Lin Tan, Guo-Xiang Wang

**Affiliations:** ^1^Department of Radiology, Anhui Provincial Hospital of Anhui Medical University, Hefei, China; ^2^Department of Radiology, The First Affiliated Hospital of University of Science and Technology of China (USTC), Division of Life Sciences and Medicine, University of Science and Technology of China, Hefei, China; ^3^Infection Hospital, The First Affiliated Hospital of University of Science and Technology of China (USTC), Division of Life Sciences and Medicine, University of Science and Technology of China, Hefei, China; ^4^Department of Interventional Ultrasound, The Second Affiliated Hospital, Anhui Medical University, Hefei, China; ^5^Department of Interventional Radiology, The First Affiliated Hospital of Bengbu Medical University, Bengbu, China; ^6^Department of Interventional Radiology, The First Affiliated Hospital of Wannan Medical College, Wuhu, China

**Keywords:** hepatocellular carcinoma, TACE, apatinib, efficacy, prognostic factors

## Abstract

**Objective:** Apatinib is a inhibitor of vascular endothelial growth factor receptor-2. To explore the efficacy and prognostic factors of transarterial chemoembolization (TACE) combined with apatinib in the treatment of Barcelona Clinic Liver Cancer stage C (BCLC C) hepatocellular carcinoma (HCC).

**Methods:** Clinical data of 146 HCC patients with BCLC stage C admitted to our hospital were collected and analyzed retrospectively, of which 76 cases were treated with TACE combined with apatinib (TACE-apatinib) and 70 with TACE alone. The tumor response, survival time, and adverse events were compared between the two groups, and the factors affecting the prognosis were analyzed.

**Results:** The objective response rate (ORR) and disease control rate (DCR) in the TACE-apatinib group were higher than in the TACE-alone group (ORR: 42.10 vs. 25.71%, *P* = 0.03; DCR: 84.21 vs. 55.71%, *P* = 0.001). The median time to progression (TTP) and overall survival (OS) in the TACE-apatinib group were longer than in the TACE-alone group (TTP: 5.5 vs. 3.7 months, *P* = 0.02; OS: 10.0 vs. 6.2 months, *P* = 0.01). Univariate and multivariate Cox regression analysis showed that tumor size, Child-Pugh class, and the presence of the portal vein tumor thrombus affect the prognosis of patients. Subgroup analysis revealed that TACE-apatinib therapy resulted in a higher OS in patients with tumor size <10 cm, without portal vein tumor thrombus, and with Child-Pugh class A (*P* < 0.05). The likelihood of adverse events (hand-foot syndrome, hypertension, oral ulcer) was significantly higher in the increased in the TACE-apatinib group than in the TACE alone group (*P* < 0.05).

**Conclusion:** TACE-apatinib is an effective and safe method for the treatment of BCLC stage C HCC. Tumor size, Child-Pugh class, and portal vein tumor thrombus affect survival time in HCC patients with BCLC stage C.

## Introduction

Hepatocellular carcinoma (HCC) is one of the most common cancers in the world with high mortality in the digestive system (1). Due to the non-specific symptoms and the occult nature of HCC, patients are usually diagnosed at the middle or late stages of the tumor, and approximately half of them have the Barcelona Clinic Liver Cancer (BCLC) stage C. In China, local treatment based on transcatheter arterial chemoembolization (TACE) is the most important therapy for patients with advanced HCC (2, 3). TACE can control tumor growth, block the blood supply to the tumor site, and induce local ischemia and hypoxia by delivering chemotherapeutic drugs and embolic agents into the feeding artery of HCC. However, studies have shown that after TACE, vascular endothelial growth factor-α (VEGF-α) is significantly increased in the residual tumor and promotes angiogenesis. Therefore, anti-angiogenic therapy is essential in patients treated with TACE (4, 5). Sorafenib is recommended as the standard treatment for BCLC stage C HCC. Sorafenib is a molecular targeted drug, which mostly inhibits tumor cell proliferation and angiogenesis. However, for patients in the Asia-Pacific region, its therapeutic effect is limited with the median overall survival (OS) at 6.5 months and the time to progression (TTP) at 2.8 months (6).

Apatinib is a small molecular targeted drug with independent intellectual property rights in China. It selectively acts on the ATP-binding site of the VEGF receptor in tumor cells, inhibiting a variety of tyrosine kinases and block its downstream pathway. These activities inhibit the migration and proliferation of vascular endothelial cells, reduce tumor neovascularization density, and inhibit residual tumor growth (7, 8). A recent randomized controlled study showed that TACE combined with apatinib is safe and can significantly prolong the overall survival and progression-free survival of patients with advanced HCC (9). Based on these findings, the clinical data of patients with HCC stage BCLC-C treated with a combination of TACE and apatinib were retrospectively evaluated.

## Materials and Methods

### Study Population

The clinical data of 146 patients with stage BCLC-C HCC treated in our hospital between January 2011 and July 2019 were analyzed retrospectively. All patients had been informed of the choice of taking apatinib and they made the decision based on their personal willingness. According to their decision of treatments, patients were classified into two groups. Seventy-six patients were included in the TACE combined with apatinib (TACE-apatinib) group, and 70 patients in the TACE alone group. Inclusion criteria were: (1) newly diagnosed liver cancer based on the criteria of the European Association of Liver Diseases; (2) patients with BCLC-C HCC; (3) Child-Pugh liver function class A or B; (4) Karnofsky score (KPS) > 60; and (5) ECOG score ≤ 2. Exclusion criteria were: (1) received systemic chemotherapy, targeted therapy, or radiofrequency ablation before entering the group; (2) contraindications for TACE; (3) severe coagulation disorder or active bleeding. Approval for this retrospective study was obtained from the Ethics Committee of the First Affiliated Hospital of USTC. Written informed consent was obtained from all of the patients before therapy.

### Treatment Methods

In both groups, TACE was performed using the modified Seldinger method. After the guide wire was inserted into the arterial sheath, the 5.0 Fr RH catheter (Cook, Bloomington, IN, USA) was placed, and dextromethorphan and palonosetron hydrochloride were injected into the abdominal aorta. After the upper derivation tube was formed, the downward catheter was inserted into the celiac trunk at the level of the middle and lower margin of the T12 vertebra. Subsequently, DSA angiography was performed to identify the hepatic artery and its branches to understand the distribution range, size, location, and the number of blood supply arteries in tumor tissue. The 3.0 Fr micro-catheter (ProgreatTM, Terumo, Tokyo, Japan) was inserted into the tumor-related blood supply artery, and lipiodol (5–15 mL; Lipiodol Ultrafluido, Guerbet, France), pirarubicin (30–50 mg) and carboplatin (30–50 mg) were injected. The specific dose depended on the embolization condition of the patient. Finally, the proximal artery was embolized with polyvinyl alcohol particles (300–500 um, Cook, USA). Some patients with severe portal vein thrombosis, wide distribution of tumors or hepatic arteriovenous fistula were not given a sufficient dose of embolic agent because of the high risk of failure of liver function recovery after treatment.

The patients in the TACE-apatinib group were treated with apatinib on the basis of TACE. Apatinib, 500 mg/day, taken orally beginning on the 3rd day after TACE. If unbearable serious adverse reactions occurred during the treatment, the dose was reduced to 250 mg daily or suspended, and patients remained under close observation and symptomatic treatment. When the adverse reactions were alleviated or disappeared, the initial dose of apatinib was gradually restored. Apatinib was discontinued 4 days before the next course of TACE and recovered 4 days after TACE. If the drug was stopped for more than a month, the patient was excluded from the study. Patients in both groups were treated until they did not tolerate the regimen or the tumor progressed; the treatment cycle was 4 weeks.

### Follow-Up and Therapeutic Evaluation

Enhanced CT or MRI, routine blood, liver and kidney function, and AFP tests were performed 1 month and 3 months after the treatment. According to The Modified Response Evaluation Criteria in Solid Tumors (mRECIST), the local curative effect was divided into four grades: complete remission (CR) if all tumor tissue disappeared on arterial phase enhanced imaging; partial remission (PR) if tumor diameter decreased by more than 30%; on arterial phase enhanced imaging; stable disease (SD) if tumor diameter on arterial phase enhanced imaging did not decrease by at least 30% or increase by at least 20%; disease progression (PD) if tumor diameter on arterial phase enhanced imaging diameter increased by at least 20% compared with the baseline value, or new lesions emerged. Objective remission rate (ORR) was calculated as (CR+PR)/total number of cases × 100%. Disease control rate (DCR) was calculated as (CR+PR+SD)/total number of cases × 100%. Adverse events were evaluated according to The Common Terminology Criteria for Adverse Events (CTCAE) version 4.0, the general terminology of adverse events.

### Study End-Points

The primary end-point of this study was the overall survival time (OS), defined as the time from the first interventional therapy to death or loss of follow-up. The secondary end-point was the time to progression (TPP), defined as the time from first interventional therapy to a definite disease progression or the time of death.

### Statistical Analysis

The SPSS 26.0 statistical software package (IBM Corp., Armonk, NY, USA) was used for statistical analysis. Median and 95% confidence interval or mean and standard deviation were used to present continuous variables, while categorical variables are presented as a frequency and percentage. For the comparison of the percentages and frequencies between the two groups, Chi-square test was performed. The survival curve was constructed using the Kaplan-Meier method, and the logarithmic rank test was performed to detect significant differences. Univariate and multivariate Cox proportional hazard regression analysis was used to identify risk factors affecting survival. All statistical tests were two-tailed, and *P* < 0.05 was considered statistically significant.

## Results

### Patient Characteristics

There was no statistical difference in general characteristics between the TACE-apatinib group and the TACE-alone group (*P* > 0.05, Table 1).

**Table 1:** Comparison of baseline characteristics between the two groups.

**Variables**	**TACE+apatinib**	**TACE**	***X* ^2^**	***P*-value**
Age (years)	55.34 ± 11.61	55.59 ± 11.68	2.162	0.90
Sex			0.05	0.81
Male	63	57		
Female	13	13		
ECOG performance status			3.12	0.07
0–1	49	35		
2	27	35		
AFP			2.79	0.09
<400	42	34		
≥400	29	41		
PVTT			2.64	0.10
No	35	23		
Yes	41	47		
Tumor size (cm)			2.79	0.09
<10	42	29		
≥10	34	41		
Number of tumors			3.09	0.07
<3	20	28		
≥3	56	42		
Extrahepatic metastasis			0.05	0.80
No	43	41		
Yes	33	29		
Presence of hepatitis			3.82	0.05
No	17	26		
Yes	59	44		
Child-Pugh class			0.56	0.45
A	42	43		
B	34	27		

*AFP, alpha fetal protein; HCC, hepatocellular carcinoma; ECOG, Eastern Cooperative Oncology Group; PVTT, portal vein tumor thrombus*.

### Comparison of Tumor Response Between the Two Treatment Groups

After 1 month of treatment, the rates of disease remission and stable disease in the TACE-apatinib group were 42.10 and 84.21%, respectively. Corresponding values in the TACE-alone group were 25.71 and 55.71% (Table 2). The difference in local curative effect between the two groups was statistically significant (*p* < 0.05).

**Table 2:** Tumor remission in two groups [n (%)].

	**CR**	**PR**	**SD**	**PD**	**ORR (%)**	**DCR (%)**
TACE+apatinib	4 (5.26%)	28 (36.84%)	32 (42.10%)	12 (15.78%)	32 (42.10%)	64 (84.21%)
TACE alone	3 (4.28%)	15 (21.42%)	21 (30.00%)	31 (44.28%)	18 (25.71%)	39 (55.71%)
*X* ^2^					4.34	14.24
*P-*value					0.03	0.001

*CR, complete response; PR, partial response; SD, stable disease; PD, progressive disease; objective response rate (ORR) = CR+PR, disease control rate (DCR) = CR +PR +SD*.

### Time to Progression and Overall Survival

**Figure 1:**
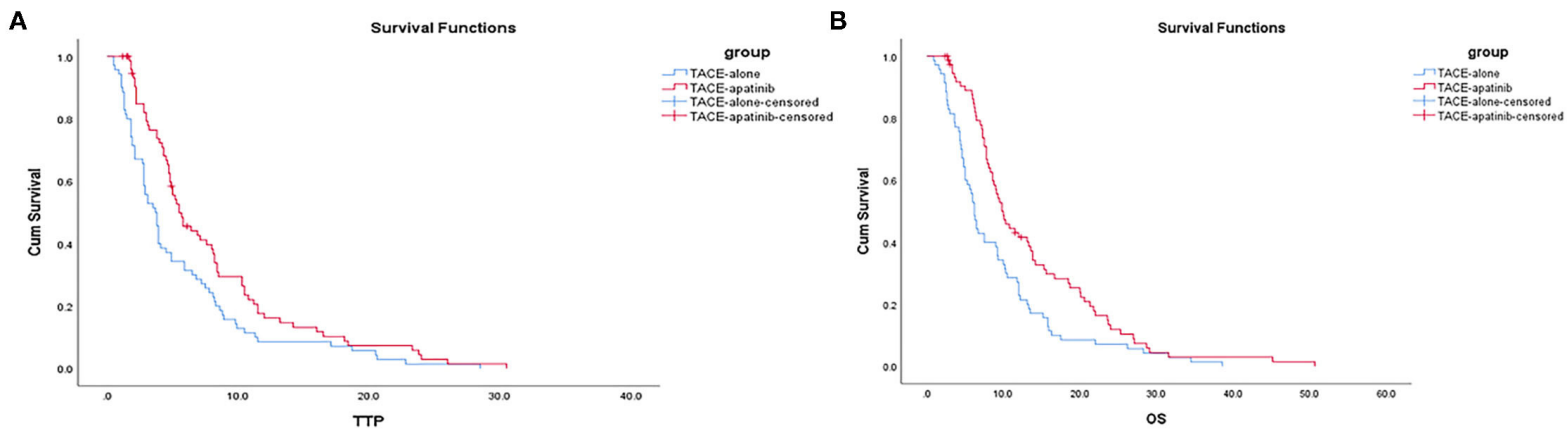
**(A)** Time to progression curves in the two groups of patients. **(B)** Overall survival curves in the two groups of patients.

The median TTP was 5.5 months (95% CI: 4.0–6.9 months) in the TACE-apatinib group and 3.7 months (95% CI: 2.9–4.4 months) in the TACE-alone group, and this difference was statistically significant (*P* < 0.02, Figure 1A). The median OS was 10.0 months (95% CI: 8.2–11.7 months) in the TACE-apatinib group and 6.2 months (95% CI: 5.4–6.9 months) in the TACE-alone group and this difference was statistically significant (*P* < 0.01, Figure 1B).

### Prognostic Factors Associated With TTP and OS

Univariate analysis showed that ECOG score, AFP, number of tumors, distant metastasis, and history of hepatitis were not related to the median OS and TTP, but portal vein tumor thrombus, tumor size, Child-Pugh grade, and treatment modality were all related to the prognosis of the patients. Cox regression multivariate analysis showed that tumor thrombus, tumor size, Child-Pugh grade, and treatment modality were independent risk factors for prognosis (*P* < 0.05, Table 3).

**Table 3:** Univariate analysis and multivariate analysis of prognosis in patients with stage BCLC-C HCC.

**Variable**	**Number**	**HR**	**95% CI**	***P*-value**	**HR**	**95% CI**	***P*-value**
Sex		0.728	(0.464–1.142)	0.167			
Male	120						
Female	26						
Age (years)		0.757	(0.533–1.074)	0.118			
≤ 50	53						
>50	93						
ECOG performance status					1.204	(0.857–1.691)	0.285
0–1	84						
2	62						
AFP(ng/ml)		1.283	(0.918–1.795)	0.145			
<400	76						
≥400	70						
PVTT		2.199	(1.549–3.122)	0.001	2.212	(1.547–3.164)	0.001
No	58						
Yes	88						
Tumor size (cm)		1.749	(1.247–2.454)	0.001	1.465	(1.026–2.092)	0.036
<10	71						
≥10	75						
Number of tumors		0.785	(0.558–1.105)	0.166			
<3	48						
≥3	98						
Extrahepatic metastasis		0.911	(0.650–1.277)	0.588			
No	84						
Yes	62						
Presence of hepatitis		0.943	(0.674–1.321)	0.734			
No	43						
Yes	103						
Child-Pugh class		1.649	(1.162–2.340)	0.005	1.542	(1.062–2.241)	0.023
A	85						
B	61						
Treatments		0.652	(0.466–0.911)	0.012			
TACE-alone	70						
TACE+apatinib	76						

*AFP, alpha fetal protein; HCC, hepatocellular carcinoma; ECOG, Eastern Cooperative Oncology Group; PVTT, portal vein tumor thrombus*.

### Subgroup Analysis

**Figure 2:**
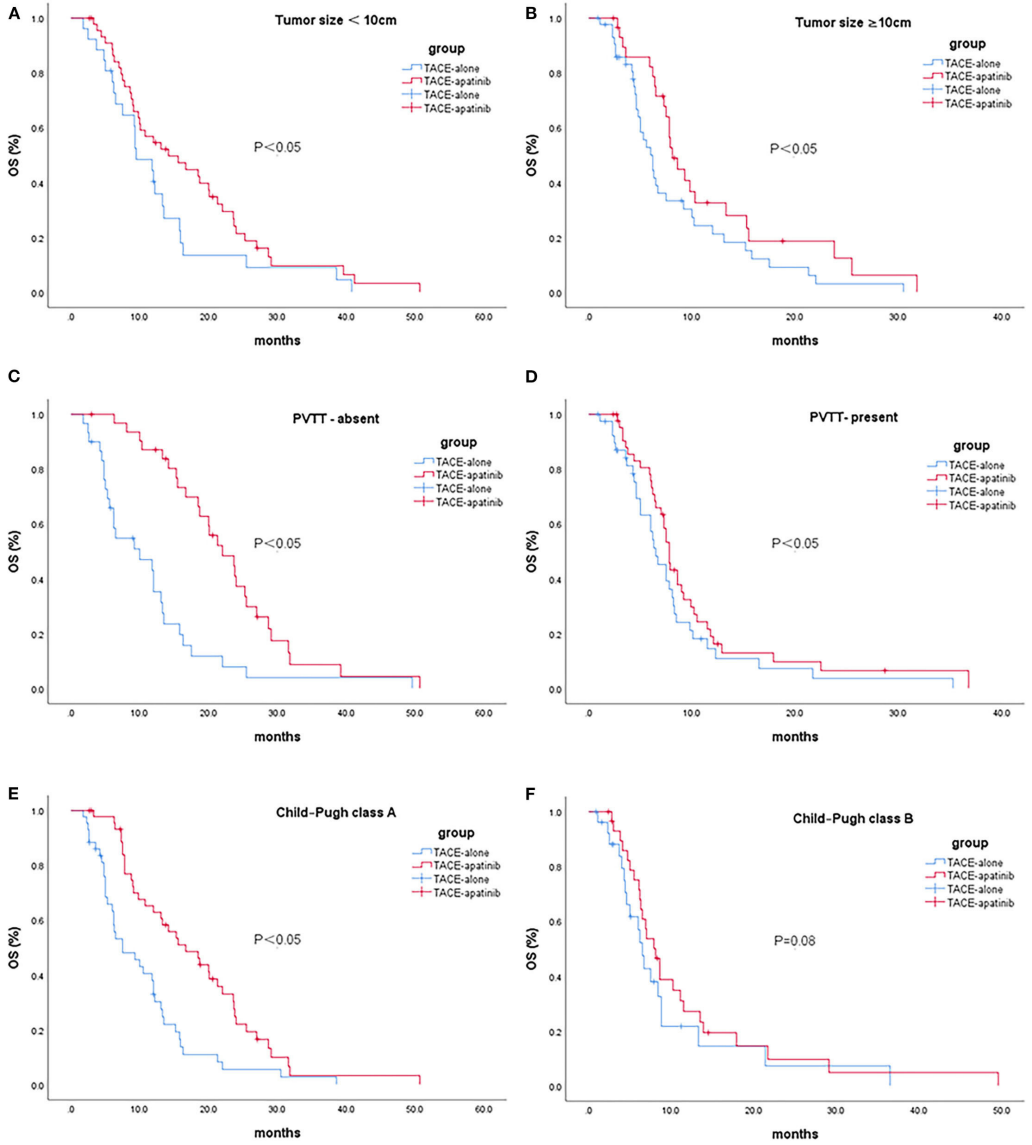
Kaplan-Meier curves showed overall survival in the TACE-apatinib and TACE-alone groups in patients with BCLC stage C HCC [**(A)** tumor size <10 cm, **(B)** tumor size ≥10 cm, **(C)** PVTT-absent, **(D)** PVTT-present, **(E)** Child-Pugh class A, and **(F)** Child-Pugh class B].

In patients with stage BCLC-C, the median OS after TACE-apatinib was higher than after TACE alone in patients with Child-Pugh class A, any tumor size, and with or without portal vein tumor thrombus (*P* < 0.05). There was no significant difference between TACE-apatinib and TACE-alone treatment in patients with Child-Pugh class B (*P* = 0.08) (Figures 2A–F).

### Adverse Events

The most frequent adverse events in the TACE-apatinib group were nausea, fever, and hand-foot syndrome, and in the TACE-alone group, nausea, fever, and fatigue. The incidence of hand and foot syndrome, hypertension, and oral ulcer in the combined treatment group was higher than in the TACE-alone group, and the difference was statistically significant (*P* < 0.05). All these adverse events disappeared after symptomatic treatment.

## Discussion

HCC patients with BCLC stage C have a vascular invasion or extrahepatic metastasis, and most of them cannot be treated surgically. Their liver function is impaired, and the prognosis is poor (10). At present, the internationally recognized treatment standard for these patients is systemic sorafenib therapy. However, due to the high price of sorafenib and possible drug resistance, it is not widely used clinically (11). In China, local interventional therapy based on TACE remains the most important treatment for HCC patients with BCLC stage C. Apatinib is an oral small-molecule targeted drug produced independently in China, which has been widely used in the treatment of gastric cancer, small cell lung cancer, esophageal cancer, and other tumors (12–14). At present, the treatment of BCLC stage C HCC with TACE-apatinib is still at the research stage. In principle, TACE can embolize the vessels supplying the tumor, promote tumor cell apoptosis, and inhibit tumor cell proliferation. TACE can rapidly reduce the tumor load of patients and improve clinical symptoms and quality of life. Apatinib can inhibit angiogenesis and vessel remodeling after TACE treatment, suppress the recurrence of the tumor and metastasis of liver cancer cells, and improve the local efficacy and long-term survival rate of patients with liver cancer (15). Therefore, these two treatments play a complementary role in the treatment of liver cancer.

In our study, the objective remission rate (ORR) and disease control rate (DCR) in the TACE-apatinib group were 42.10 and 84.21%, respectively. These values were higher than those of TACE combined with sorafenib or sorafenib alone. This difference reflects the fact that apatinib, a receptor tyrosine kinase inhibitor selectively targeting VEGFR-2, has a binding affinity 10 times higher than sorafenib (16). Apatinib is generally well-tolerated and is associated with controllable adverse reactions; the most common drug-related adverse reactions are hand and foot syndrome, hypertension, and albuminuria, which is similar to the previously reported study of apatinib as the only treatment and TACE combined with apatinib (17, 18). In the present study, the incidence of adverse reactions, particularly hypertension, hand and foot syndrome, and oral mucositis, was higher in the TACE-apatinib group than in the TACE-alone group. There was no significant difference in post-embolization syndrome between the two groups.

Recent clinical studies have shown that TACE combined with apatinib can prolong the survival of patients with advanced liver cancer (19, 20). The current analysis showed that for HCC patients with BCLC stage C, the median survival time in the TACE-apatinib group was 3.8 months longer than in the TACE-alone group, and the difference was statistically significant (*P* < 0.05). The median TTP in the TACE-apatinib group was 5.5 months, and in the TACE-alone group, 3.7 months, indicating that the combined therapy could delay tumor progression and reduce tumor load. Some studies have confirmed that the median survival time of BCLC stage C HCC patients treated with TACE alone is 7.1 months and in patients treated with supportive therapy is 5.1 months, indicating that TACE can significantly prolong the survival time of patients (21). Our work documented that the median survival time in the TACE-apatinib group was 10.0 months, implying that the combined therapy further improved the survival time of patients with BCLC stage C HCC. From the point of view of clinical practice, TACE combined with apatinib can significantly prolong the survival time of patients with liver cancer.

The conducted univariate and multivariate analysis demonstrated that tumor size, Child-Pugh class, and portal vein tumor thrombus were independent prognostic factors for the survival of patients with BCLC stage C HCC. Earlier studies documented that the 1- and 3-year survival rate of patients with small liver cancer treated with TACE is significantly longer than that of patients with large liver cancer. Tumor size also significantly affects the prognosis of primary liver cancer treated with the combination of TACE and apatinib; with the increase in tumor size, the median survival time of patients was shortened (22). Current studies have confirmed that the larger the tumor size and the higher the activity, the more difficult it is to completely inhibit tumor growth, and the easier it is to relapse and metastasize, negatively affecting the prognosis of patients (23, 24). Liver function grade was also shown to be related to prognosis. The main reason underlying this relationship is that the higher grade of liver function is associated with a worse compensatory ability of liver function, lower liver tolerance, and poor conditions for TACE re-treatment, which can not only fail to provide a good effect but may even accelerate liver failure in some patients (25, 26). Although portal vein tumor thrombus is present in a considerable number of HCC patients with BCLC stage C, this study shows that cancer thrombus is still an independent risk factor for the prognosis of patients. The portal vein is mainly derived from the tumor, so portal vein tumor thrombus is more common. Tumor thrombus in the portal vein system can not only cause intrahepatic or extrahepatic metastasis of the tumor but can also block the portal vein system, resulting in an increased portal vein pressure. Additionally, it can cause dilatation and tortuosity of the portal vein system, especially in the lower segment of the esophagus and gastric fundus vein, increasing the probability of upper gastrointestinal bleeding (27). As the normal liver tissue is primarily supplied by the portal vein, portal hypertension will reduce the effective blood supply to the liver tissue and lead to further impairment of liver function.

This study has some limitations. The number of cases is relatively small, and the follow-up time is relatively short. Moreover, this is an observation and retrospective study, and thus may be affected by subjective selection bias. More large-scale multicenter prospective studies are needed to verify the efficacy of the combination of TACE and apatinib.

In conclusion, TACE combined with apatinib is a safe and effective treatment of advanced liver cancer and may represent a novel and feasible treatment modality for BCLC stage C HCC.

## Data Availability Statement

The original contributions presented in the study are included in the article/supplementary material, further inquiries can be directed to the corresponding author.

## Ethics Statement

The studies involving human participants were reviewed and approved by the First Affiliated Hospital of USTC (Anhui Provincial Hospital) Medical Research Ethics Committee. The patients/participants provided their written informed consent to participate in this study.

## Author Contributions

W-FL conceived the study and wrote the manuscript. DL, X-HZ, BJ, Y-LT, and G-XW collected the data. SL and K-CL performed statistical analyses. SL and W-FL provided support and helped with manuscript revision. All authors were involved in analyzing the results. All authors contributed to the article and approved the submitted version.

## Funding

This work was supported by the Fundamental Research Funds for the Central Universities (No. WK9110000061) and by the Anhui Natural Science Foundation (No. 1808085MH254).

## Conflict of Interest

The authors declare that the research was conducted in the absence of any commercial or financial relationships that could be construed as a potential conflict of interest.

## Publisher's Note

All claims expressed in this article are solely those of the authors and do not necessarily represent those of their affiliated organizations, or those of the publisher, the editors and the reviewers. Any product that may be evaluated in this article, or claim that may be made by its manufacturer, is not guaranteed or endorsed by the publisher.
